# Predicting Uptake of the COVID Coach App Among US Military Veterans: Funnel Analysis Using a Probability-Based Panel

**DOI:** 10.2196/36217

**Published:** 2022-04-05

**Authors:** Beth K Jaworski, Katherine Taylor, Kelly M Ramsey, Adrienne J Heinz, Sarah Steinmetz, Jason E Owen, Jack Tsai, Robert H Pietrzak

**Affiliations:** 1 National Center for Posttraumatic Stress Disorder Dissemination & Training Division US Department of Veterans Affairs Menlo Park, CA United States; 2 National Center on Homelessness Among Veterans US Department of Veterans Affairs Tampa, FL United States; 3 School of Public Health San Antonio Campus University of Texas Health Science Center at Houston San Antonio, TX United States; 4 National Center for Posttraumatic Stress Disorder VA Connecticut Healthcare System US Department of Veterans Affairs New Haven, CT United States; 5 Department of Psychiatry Yale School of Medicine New Haven, CT United States

**Keywords:** COVID-19, coronavirus, mobile app, mHealth, digital health, mental health, public mental health, stress, coping, older adults, veterans

## Abstract

**Background:**

Although the COVID-19 pandemic has not led to a uniform increase of mental health concerns among older adults, there is evidence to suggest that some older veterans did experience an exacerbation of preexisting mental health conditions, and that mental health difficulties were associated with a lack of social support and increasing numbers of pandemic-related stressors. Mobile mental health apps are scalable, may be a helpful resource for managing stress during the pandemic and beyond, and could potentially provide services that are not accessible due to the pandemic. However, overall comfort with mobile devices and factors influencing the uptake and usage of mobile apps during the pandemic among older veterans are not well known. COVID Coach is a free, evidence-informed mobile app designed for pandemic-related stress. Public usage data have been evaluated; however, the uptake and usage of the app among older veterans have not been explored.

**Objective:**

The purpose of this study was to characterize smartphone ownership rates among US veterans, identify veteran characteristics associated with downloading and use of COVID Coach, and characterize key content usage within the app.

**Methods:**

Data were analyzed from the 2019-2020 National Health and Resilience in Veterans Study (NHRVS), which surveyed a nationally representative, prospective cohort of 3078 US military veterans before and 1 year into the pandemic. The NHRVS sample was drawn from KnowledgePanel, a research panel of more than 50,000 households maintained by Ipsos, Inc. The median time to complete the survey was nearly 32 minutes. The research version of COVID Coach was offered to all veterans who completed the peripandemic follow-up assessment on a mobile device (n=814; weighted 34.2% of total sample). App usage data from all respondents who downloaded the app (n=34; weighted 3.3% of the mobile completers sample) were collected between November 14, 2020, and November 7, 2021.

**Results:**

We found that most US veterans (81.5%) own smartphones, and that veterans with higher education, greater number of adverse childhood experiences, higher extraversion, and greater severity of pandemic-related posttraumatic stress disorder symptoms were more likely to download COVID Coach. Although uptake and usage of COVID Coach were relatively low (3.3% of eligible participants, n=34), 50% of the participants returned to the app for more than 1 day of use. The interactive tools for managing stress were used most frequently.

**Conclusions:**

The COVID-19 pandemic has increased the need for and creation of digital mental health tools. However, these resources may require tailoring for older veteran populations. Future research is needed to better understand how to optimize digital mental health tools such as apps to ensure uptake and usage among older adults, particularly those who have experienced traumas across the lifespan.

## Introduction

### Background

#### Mental Health Impact of the COVID-19 Pandemic Among Older Veterans

The coronavirus pandemic has taken an extraordinary toll on health and well-being globally. In the United States, over 900,000 people have died due to COVID-19. Vaccines against the coronavirus are now available to anyone over the age of 5 years in the United States. However, distribution has been uneven within and across states, and many individuals have expressed unwillingness to be vaccinated [1]. The lack of vaccination prolongs the pandemic and in turn its negative impact on society. In addition to physical health consequences, the pandemic and its mitigation strategies have led to a host of negative mental health consequences, including increased symptoms of anxiety, depression, posttraumatic stress disorder (PTSD), psychological distress, increased substance use to cope with pandemic-related stressors, and increased suicidal ideation [2-5]. Unhealthy alcohol use, increased tobacco and cannabis use, and potential misuse of prescription opioids and benzodiazepines were identified as key areas of clinical concern for mental health providers [6]. Recent evidence suggests that these concerns were well-founded, as drug overdose deaths rose 27.2% between April 2020 and April 2021 [7].

However, research indicates a more nuanced, multifaceted relationship between age and pandemic-related mental health concerns [8]. Researchers have documented a range of pandemic impacts on mental health, including negative outcomes, no change in mental health symptomology, and even some evidence of mental health gains. For example, among a sample of older adults with chronic PTSD, researchers did not find that the pandemic significantly increased adverse mental health outcomes, and in fact found that PTSD symptoms among individuals with a PTSD diagnosis decreased relative to those of a trauma-exposed comparison group [9]. Additionally, some veterans have demonstrated increased posttraumatic growth (PTG) and resilience associated with the pandemic. In a probability-based sample of older US military veterans, Pietrzak and colleagues [10] found that 43.3% of the sample endorsed PTG, and veterans who screened positive for COVID-19–associated PTSD were more likely to endorse all aspects of PTG compared to veterans who screened negative for COVID-19–associated PTSD symptoms.

Although the pandemic may not have led to a uniform increase of mental health concerns among older adults, those experiencing loneliness and social isolation may have been particularly vulnerable to increased psychological distress and an exacerbation of preexisting mental health conditions. Loneliness is often a concern among older adults, particularly among individuals with low socioeconomic status [11], and social isolation and loneliness are associated with increased morbidity and mortality [12], even outside the context of a global pandemic. Older veterans indicated that the pandemic increased loneliness and sorrow due to the isolation and disruption of their ordinary routines [13], and researchers also found that a lack of social support and increasing numbers of pandemic-related stressors were associated with mental health difficulties [14]. In a national sample of US veterans, the pre- to peripandemic prevalence of generalized anxiety disorder increased from 7.1% to 9.4%, with the most pronounced increase observed in veterans aged 45-64 years (8.2% to 13.5%) [15]. Results of this study further indicated that prepandemic loneliness and pandemic-related social stressors were associated with an increase in psychological distress [15]. Although suicide among veterans over the course of the pandemic did not increase, low social support and worsening of social relationships were among the risk factors present for veterans who did develop new-onset suicide ideation during the pandemic [16]. Additionally, at the beginning of the pandemic, it was hypothesized that there might be a mental health crisis among older adults due in part to complications from the difficulty in adopting technologies useful during quarantine (eg, software to facilitate telehealth visits or stay connected with loved ones) and lack of contact with friends, family, and caregivers [8]. Evaluation of video mental health visits among US veterans during the COVID-19 pandemic suggests this may be the case. Researchers found that older patients and those with low socioeconomic status had lower odds of completing greater than 50% of their visits via video compared to in-person visits or phone calls [17]. Taken together, these findings suggest that older veterans with low socioeconomic status, and those experiencing loneliness and social isolation were more likely to face negative mental health consequences of the pandemic. Thus, promoting positive social connections and utilizing effective coping strategies for aging veterans have been identified as key suggestions for helping with pandemic-related stressors [18].

#### Potential of Mental Health Apps to Support Older Veterans

Very early in the pandemic, public health scholars called for prevention and early intervention efforts to help promote individual and population mental health [19]. Digital mental health options have been identified as one possible solution with great potential for the pandemic and beyond [20]. However, an important precursor to the adoption of digital mental health technologies, and particularly mental health apps, is mobile device ownership. Although older adults in the United States may be somewhat less likely than their younger counterparts to use mobile devices, the majority (61%) own smartphones [21]. Older adults express interest in using mobile devices to support health [22], and in some cases their engagement with digital health products may exceed that of younger adults [23]. In general, prior research suggests that older adults are interested in apps for health, but uptake and usage continue to be relatively low [24]. Among older veterans, many are interested in apps to support health [25], but tend to have mixed opinions about mental health apps based on sociodemographics such as rurality [26]. Connolly and colleagues [17] found that rural veterans are more likely to oppose app usage, describe smartphones as hard to navigate, and cite barriers to usage compared to urban-dwelling populations. However, educational materials and training programs can be successfully implemented to increase older veterans’ comfort with using apps and overcome barriers to utilization [27,28]. With additional efforts to promote comfort with and confidence in mental health apps, they may be a scalable, accessible, and helpful resource for older veterans.

#### Need for Studying COVID Coach Among Older Veterans

To address the mental health needs of veterans in the wake of the pandemic, the US Department of Veterans Affairs (VA) rapidly implemented several virtual resources and practices to support telemental health [29]. In addition to resources for the delivery of telemental health care, the VA’s National Center for PTSD also created COVID Coach, a free public mental health app designed to help individuals manage stress and anxiety resulting from the COVID-19 pandemic. Mobile and internet-based interventions, including COVID Coach, have been identified as a population-level, primary prevention resource for pandemic-related mental health impacts [30]. The app was created in 6 weeks and released on both the Android and iOS platforms at the end of April 2020. Between the app launch and the end of October 2021, the app has been downloaded over 200,000 times (Android, n=27,082 downloads; iOS, n=188,224 downloads).

The COVID Coach app contains four key content areas: (1) Manage Stress (interactive coping tools); (2) Learn (psychoeducational topics covering ways to stay well, stay balanced, navigate relationships, stay safe, and stay healthy from COVID-19); (3) Mood Check (for tracking personal goals and tracking well-being [Warwick Edinburgh Mental Well-Being Scale], and symptoms of anxiety [Generalized Anxiety Disorder-7 questionnaire, GAD-7], depression [Patient Health Questionnaire-9, PHQ-9], and PTSD [PTSD Checklist for DSM-5, PCL-5]); and (4) Find Resources (a comprehensive repository of resources and supports across a range of topics, including mental health crisis support, ways to meet basic needs, and local information about COVID-19). Screenshots of COVID Coach are provided in Multimedia Appendix 1. COVID Coach has been well-received in the Apple App Store (average star rating=4.8 out of 5; n=871 ratings) and Google Play Store (average star rating=4.8 out 5; n=308 ratings). An initial evaluation of the anonymous public usage data revealed that app users have primarily utilized the “Manage Stress” section of the app (with interactive and audio-guided tools for coping with stress and anxiety), but that collectively, thousands of users have accessed the psychoeducational information, assessments, and resources [31]. However, similar to many other mental health apps, engagement and retention were low (eg, [32]). Additionally, understanding for whom and under what conditions COVID Coach was utilized was not possible because usage data from the publicly available version of the app are completely anonymous.

Several factors may be associated with the decision to download and use mobile mental health apps during the COVID-19 pandemic. These include sociodemographic characteristics such as age and education [33]; personality characteristics such as extraversion and conscientiousness [34]; preexisting mental health difficulties such as trauma exposure [35]; event-specific stressors such as pandemic-related social restriction stress [31]; and characteristics of the app itself, such as usability, perceived utility, and trustworthiness [36]. To date, however, no known study has examined factors associated with US military veterans’ uptake and usage of mobile apps specific to addressing pandemic-related stressors, such as COVID Coach.

### Current Study

There are estimated to be 19 million US veterans, which accounts for approximately 10% of the US population [37]. Gulf War–era veterans comprise the largest segment, followed by Vietnam-era veterans [38]. Thus, older veterans represent a significant proportion of the overall veteran population [39], and many are open to exploring the usage of mental health apps [25,26,40]. To explore smartphone ownership as well as the uptake and usage of an app for pandemic-related stress and anxiety specifically among older veterans, this study was guided by the following four aims: (1) understand the relationship between sociodemographic characteristics and survey completion on a mobile device compared to a laptop or desktop computer; (2) identify sociodemographic, prepandemic, and pandemic-related variables associated with downloading COVID Coach; (3) explore differences among veterans who used the app for only 1 day compared to those who returned to the app for 2 or more days; and (4) characterize key content usage within the app.

## Methods

### Study Design and Participants

Data were analyzed from the 2019-2020 National Health and Resilience in Veterans Study (NHRVS), which surveyed a nationally representative, prospective cohort of US military veterans. The NHRVS sample was drawn from KnowledgePanel, a research panel of more than 50,000 households maintained by Ipsos, Inc. KnowledgePanel is a probability-based, online, nonvolunteer access survey panel of a nationally representative sample of US adults that covers approximately 98% of US households. To permit generalizability of the study results to the entire population of US veterans, Ipsos statisticians computed poststratification weights using the benchmark distributions of the following sociodemographic characteristics of US military veterans from the most recent (August 2019) Current Veteran Population Supplemental Survey of the US Census Bureau’s American Community Survey [41]: age, gender, race/ethnicity, Census Region, metropolitan status, education, household income, branch of service, and years in service. With exception of the app usage data, all percentages and inferential statistics displayed in the Results section reflect weighted percentages, utilizing these poststratification weights.

A total of 4069 veterans completed the prepandemic survey (median completion date November 21, 2019) prior to the first documented COVID-19 cases in the United States and 3078 (75.6%) completed a 1-year peripandemic follow-up assessment (median completion date November 14, 2020). The median completion time was 31.9 minutes (IQR 19.9 minutes). These veterans were 22 to 99 years old (mean age 63.2, SD 14.7 years). The sample was mostly male (91.6%); 79.3% were non-Hispanic Caucasian, 10.3% non-Hispanic African American, 6.0% Hispanic, and 4.4% bi/multiracial or other racial identity. The sample included all branches of the US military (majority Army [47.3%], Navy [20.8%], or Air Force [18.9%]), 35.0% were combat veterans, 79.6% reported having enlisted in the military, and 20.1% reported utilizing VA as their primary source of health care.

**Figure 1:**
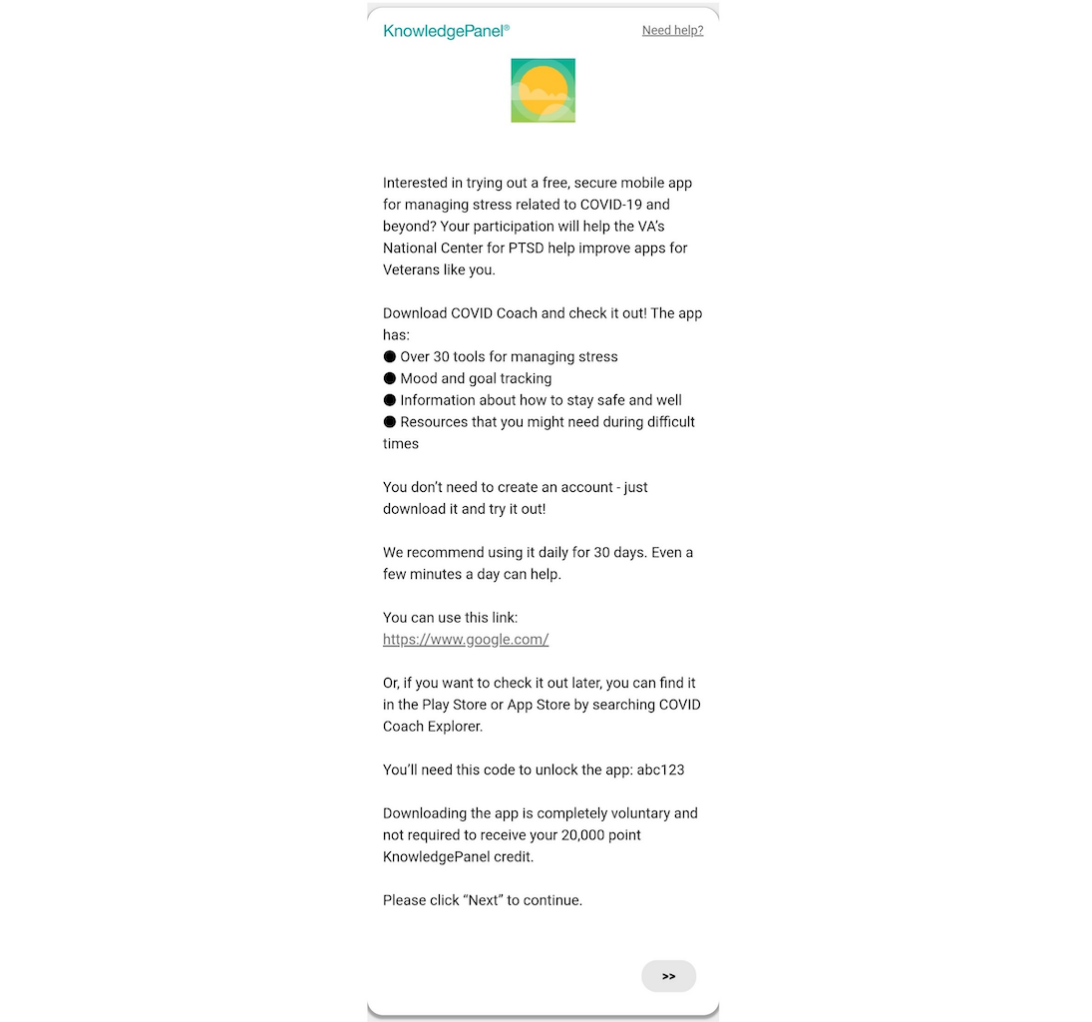
Invitation to download COVID Coach Explorer.

A total of 814 (34.2%) of the sample completed the survey on a tablet or smartphone and were eligible to download the research version of the COVID Coach app. On the final page of the online questionnaire, these participants saw a brief description of COVID Coach, as well as a link to download the app (see Figure 1 for the message and format of the invitation). Of this sample, 34 (3.3%) downloaded the COVID Coach app. Relative to veterans who did not download the app, those who did spent significantly more time on the landing page (mean 118.8, SD 107.7 seconds vs mean 33.8, SD 49.7 seconds; t_812_=9.10, *P*<.001), but did not differ with respect to whether they used devices with iOS (52.9% vs 46.5%) or Android (47.1% vs 53.5%) operating systems (*χ*^2^_1_=0.55, *P*=.46).

### Ethical Considerations

All participants provided informed consent and the Human Subjects Committee of the VA Connecticut Healthcare System approved the study. Prior to completing the survey, Ipsos obtains informed consent from all participants to participate in the NHRVS, and participants received 15,000 points (equivalent to US $15) as compensation for their participation in this study.

### COVID Coach Explorer and Mobile Analytics Data

The research-enabled version of COVID Coach (COVID Coach Explorer, version 1.0) is available for Android [42] and iOS [43]. COVID Coach Explorer contains the exact same features and content as COVID Coach, which includes interactive, evidence-informed tools for coping with stress and anxiety; information about how to stay well, stay connected, and navigate challenges; self-monitoring mental health symptoms and goals; and resources to discover and connect with various types of verified and vetted support. However, COVID Coach Explorer allows researchers to assign a unique code to each research participant, and the app usage data can only be associated with the participant identifiers by approved members of the research team. The app only collects information about app use such as screens selected, button presses, and other nonidentifying patterns, but these usage data are only linked to the unique alphanumeric code. Fully nonidentifying and encrypted event sequences were stored using JavaScript object notation (JSON) format on a remote GovCloud server that meets VA security and privacy requirements. Data are accessible from VA App Connect software, which has been approved for use under the VA’s Technical Reference Model [44]. Each in-app event contains a timestamp (in Coordinated Universal Time) that corresponds to when the event actually occurred, but data are only transmitted to the server when the app is in use and connected to WiFi or utilizing a data plan.

For the purpose of this study, mobile analytics data with timestamps between November 14, 2020, and November 7, 2021, were extracted from the research server on November 7, 2021. Between November 14, 2020, and November 7, 2021, 1752 in-app related events were captured (Android, n=1063; iOS, n=689) across the 34 participants who downloaded COVID Coach Explorer (Android, n=16; iOS, n=18).

### Measures

#### Survey Assessments

The questionnaire administered to panelists included a range of assessments, including demographic information (eg, age, gender, education, military branch, mobile device ownership), prepandemic psychosocial risk factors (eg, adverse childhood experiences, PTSD symptoms, alcohol use disorder symptoms), changes in psychosocial risk factors from pre- to peripandemic, COVID-19 infection stressors, and COVID-19 pandemic stressors. For a detailed overview of each instrument, please see Table 1.

**Table 1:** Measures of psychiatric, sociodemographic, military, and psychosocial variables and COVID-19 infection and pandemic stressors.

Characteristics		Measures
Sociodemographic characteristics		Age (continuous), sex (male, female), race/ethnicity (white, nonwhite), education (college graduate or higher, up to high school diploma), marital status (married/living with partner, not), household income (US $60,000 or more, less than $60,000), employment status (working, not), region of country (south, west, midwest, northeast), metropolitan versus nonmetropolitan residence
Military characteristics		Combat veteran status (combat exposure, not), military branch (Army, Navy, Air Force, Marine Corps, other); combat veteran status (yes, no), years of military service
**Prepandemic psychosocial risk factors**		
	Adverse childhood experiences	Adverse Childhood Experiences Questionnaire (ACEQ) score [45]
	Total traumas	Items endorsed on Life Events Checklist for DSM-5^a^ (LEC-5) [46]
	Lifetime MDD^b^ or PTSD^c^	Lifetime MDD was assessed according to DSM-5 diagnostic criteria using the Mini International Neuropsychiatric Interview (MINI) [47]. Lifetime PTSD was defined as a score of 33+ [48] on the PCL-5^d^ [49], which was modified to include lifetime ratings of all PTSD symptoms in relation to veterans’ self-reported “worst” Criterion A trauma on the LEC-5 [46]. Veterans who met criteria for either disorder were coded positive for lifetime MDD or PTSD
	Lifetime AUD^e^ or DUD^f^	Lifetime AUD and DUD were defined as meeting DSM-5 diagnostic criteria for AUD or DUD, respectively, as assessed using the MINI [47]; veterans who met criteria for either disorder were coded positive for lifetime AUD or DUD
	Number of medical conditions	Sum of number of medical conditions adapted from the Alcohol Use Disorders and Associated Disabilities Interview Schedule [50]: “Has a doctor or health care professional ever told you that you have any of the following medical conditions?” (eg, arthritis, cancer, diabetes, heart disease, asthma, kidney disease); range 0-24 conditions
	Personality characteristics	Ten-Item Personality Inventory (TIPI) [51] scores, which yield measures of the “Big 5” personality constructs of extraversion, agreeableness, conscientiousness, emotional stability, and openness to experiences
COVID-19 infection stressors		COVID-19 infection status (endorsement of self infected, know someone in household who was infected, know someone not in household who was infected, and know someone who died of COVID-19)
COVID-19 pandemic stressors		Questions from the National Institute of Mental Health Coronavirus Health Impact Survey [52] were used to assess COVID-19–associated worries and concerns at the peripandemic assessment. Factor analysis revealed that these items loaded on five factors (eigen values=1.01-4.94): COVID-19–related disease worries (eg, “In the past month, how worried have you been about being infected with coronavirus?”); COVID-19 social restriction stress (eg, “How stressful have these changes in social contacts been for you?”); COVID-19–associated socioeconomic stress (eg, “In the past month, to what degree have changes associated to the pandemic created financial problems for you or your family?”); COVID-19–associated relationship difficulties (eg, “Has the quality of the relationships between you and members of your family changed?”); and COVID-19–associated social engagement (eg, “In the past month, how many people, from outside of your household, have you had an in-person conversation with?”).

^a^DSM-5: Diagnostic and Statistical Manual of Mental Disorders, 5th edition.

^b^MDD: major depressive disorder.

^c^PTSD: posttraumatic stress disorder.

^d^PCL-5: PTSD Checklist for DSM-5.

^e^AUD: alcohol use disorder.

^f^DUD: drug use disorder.

#### App Use Metrics

App use metrics are similar to those outlined in the evaluation of the public use (“in the wild”) data [31]. Overall frequencies for key content usage were computed for each of the four key sections in the app: *Manage Stress* (tried a tool), *Learn* (viewed a learn topic), *Mood Check* (created and rate a goal or completed an assessment), and *Find Resources* (viewed at least one specific subsection within Find Resources). Because of the variability within and across mobile device operating systems with respect to reliably in capturing unique app sessions (eg, [31,53]), we decided to assess frequency of use by unique days of use rather than by sessions or visits. Distinct days of use within the observation window were calculated, as well as retention days (the number of days between the first day of use and last day of use). Activities within each of the key content areas were totaled as well as tabulated by each distinct day of use. 

### Analyses

#### Overview

Broadly, we utilized a funnel or conversion analytic strategy (eg, [54,55]) to characterize how many veterans tried the COVID Coach app, among those who downloaded it, from within the population of veterans who were offered the opportunity to download it.

#### Survey Data

Data analyses proceeded in five steps. First, we performed independent-samples *t* tests and *χ^2^* analyses to compare sociodemographic characteristics of veterans who did and did not complete the survey on a smartphone or tablet. Second, we performed independent-samples *t* tests and *χ^2^* analyses to compare sociodemographic, prepandemic, and pandemic-related variables in the subset of veterans who completed the survey on a smartphone or tablet and between those who did and did not download/use the COVID Coach app. Third, we performed multivariable binary regression analyses to identify sociodemographic, prepandemic, and pandemic-related variables that were independently associated with (1) completing the survey on a device compared to a desktop or personal computer and (2) downloading the COVID Coach app. Variables that were associated with completing the survey on a device versus desktop or personal computer and downloading the app in bivariate analyses (*P*<.05) were entered into these analyses. Planned secondary logistic regression analyses were performed to identify aspects of multidimensional measures (eg, PTSD symptoms) that were associated with downloading the app. Finally, to compare veterans who used the app once versus more than once, we performed independent-samples *t* tests and *χ^2^* analyses; these analyses were unweighted.

#### App Usage Data

SQLPro Studio (Hankinsoft Development, Inc) was used for all data preprocessing and extraction. SAS OnDemand for Academics (SAS Institute, Cary, NC) was used for statistical analyses of the app usage data. We calculated descriptive statistics for key content usage, unique days of app use, and retention. *χ^2^* analyses were performed to understand differences in returning to the app for a second day of use based on key content usage on the first day of app use. We ran separate *χ^2^* analyses for each predictor.

## Results

### Smartphone Ownership and Survey Completion Overview

Overall, the majority of participants (n=2262, 65.84%) completed the survey on a desktop or personal computer. Notably, regardless of the device type used to complete the survey, many veterans reported owning a smartphone (n=2443, 81.50%). Desktop or personal computer completers differed significantly across a number of dimensions compared to those who completed the survey on a mobile device (smartphone or tablet). Participants who completed the survey on a mobile device tended to be younger, identify as female or nonwhite race/ethnicity, be currently employed, and live in a household with an annual income of US $60,000 or greater compared with those who completed the survey on a desktop or personal computer. They were also more likely to have lifetime histories of major depressive disorder or PTSD and alcohol use disorder or drug use disorder, but reported fewer medical conditions. Importantly, the majority of participants indicated owning a smartphone (83.0% among veterans who did not complete the survey on a mobile device and 94.6% among veterans who completed their survey on a mobile device). See Table 2 for additional information about participant characteristics.

The multivariable logistic regression analysis revealed that lower age (odds ratio [OR] 0.97, 95% CI 0.96-0.97), female gender (OR 2.10, 95% CI 1.62-2.74), nonwhite race/ethnicity (OR 1.74, 95% CI 1.43-2.11), lower than college education (OR 1.53, 95% CI 1.27-1.85), and higher household income (OR 1.34, 95% CI 1.12-1.61) were independently associated with completing the survey on a mobile device, while residing in the west of the country was associated with a lower likelihood of doing so (OR 0.60, 95% CI 0.47-0.76). None of the other variables differentiated these groups (all *P*>.05).

**Table 2:** Characteristics of US veterans by device type used for survey completion.

Characteristic			Completed survey on a desktop or personal computer (n=2264; weighted 65.8%)		Completed survey on a mobile device (n=814; weighted 34.2%)		*t* (*df*=3070) or *χ*^*2*^		*P* value
Age, weighted mean (SD)			64.8 (14.6)		55.5 (15.3)		16.53		<.001
Female gender, n (weighted %)			183 (6.0)		161 (17.0)		92.28 (*df*=1)		<.001
**Race/ethnicity, n (weighted %)**							71.11 (*df*=3)		<.001
	White, non-Hispanic	1932 (83.5)		609 (70.5)					
	Black, non-Hispanic	131 (8.7%)		81 (13.7%)					
	Hispanic	130 (4.3)		86 (9.6)					
	Bi/Multiracial or Other	71 (3.6)		38 (6.3)					
College graduate or higher education, n (weighted %)			1072 (33.9)		336 (28.4)		9.47 (*df*=1)		.002
Married/partnered, n (weighted %)			1634 (73.8)		590 (75.0)		0.54 (*df*=1)		.46
Currently employed, n (weighted %)			787 (45.0)		408 (61.2)		72.55 (*df*=1)		<.001
Annual household income US $60,000 or above, n (weighted %)			1328 (60.3)		525 (64.4)		4.96 (*df*=1)		.03
**Region of country, n (weighted %)**							31.60 (*df*=3)		<.001
	South	835 (38.9)		353 (47.6)					
	West	597 (25.2)		174 (17.9)					
	Midwest	528 (20.8)		187 (21.8)					
	Northeast	304 (15.1)		100 (12.7)					
Non-metro residence			331 (15.8)		143 (17.5)		1.41		.24
**Military branch, n (weighted %)**							10.93 (*df*=4)		.03
	Army	900 (47.9)		298 (45.3)					
	Navy	467 (19.5)		206 (21.2)					
	Air Force	557 (19.5)		176 (17.8)					
	Marine Corps	128 (4.9)		61 (7.5)					
	Other	212 (8.2)		73 (8.2)					
Combat veteran, n (weighted %)			781 (33.3)		271 (37.5)		5.60 (*df*=1)		.02
10+ years of military service, n (weighted %)			809 (35.0)		323 (39.5)		6.23 (*df*=1)		.01
**Health variables, n (weighted %)**									
	Lifetime MDD^a^ and/or PTSD^b^	356 (16.7)		215 (27.8)		51.83 (*df*=1)		<.001	
	Lifetime AUD^c^ and/or DUD^d^	924 (41.0)		343 (48.2)		14.16 (*df*=1)		<.001	
	Number of medical conditions, weighted mean (SD)	2.9 (2.2)		2.7 (2.0)		3.02		.001	
Own any type of cell phone, n (weighted %)			2,103 (91.9%)		793 (97.2%)		32.87 (*df*=1)		<.001
Own a smartphone, n (weighted %)			1,698 (76.6%)		745 (91.1%)		96.55 (*df*=1)		<.001

^a^MDD: major depressive disorder.

^b^PTSD: posttraumatic stress disorder.

^c^AUD: alcohol use disorder.

^d^DUD: drug use disorder.

### Predicting COVID Coach Explorer Download

Table 3 shows characteristics for veterans who did and did not download the COVID Coach app. Bivariate analyses revealed that, relative to those who did not download the COVID Coach app, those who did were more likely to have completed college or higher education reported more adverse childhood experiences and potentially traumatic events and scored higher on a measure of extraversion. They also reported a greater number of hours of daily exposure to pandemic-related media and greater severity of pandemic-related worsening of relationships and PTSD symptoms.

Results of the multivariable binary logistic regression analyses revealed that college graduate or higher education (OR 3.67, 95% CI 1.73-7.79), greater number of adverse childhood experiences (OR 1.22, 95% CI 1.05-1.41), higher extraversion (OR 1.68, 95% CI 1.31-2.14), and greater severity of pandemic-related PTSD symptoms (OR 2.46, 95% CI 1.10-5.53) were associated with downloading the COVID Coach app. None of the other variables was significant (all *P*>.19). A posthoc analysis of COVID-related PTSD symptoms revealed that greater severity of exaggerated startle symptoms drove the association with downloading/using the COVID Coach App (OR 1.62, 95% CI 1.11-2.36); none of the other PTSD symptoms was significant (all *P*>.14).

**Table 3:** Characteristics of US veterans who completed the survey on a tablet or smartphone and did and did not download the COVID Coach app.

Characteristic		*Did not* download COVID Coach (n=780, weighted 65.8%)	Downloaded COVID Coach (n=34, weighted 3.3%)	*t* (*df*=1049) or *χ*^*2*^		*P* value	
Age (years), weighted mean (SD)		55.6 (15.3)	51.8 (15.5)	1.44		.08	
Female gender, n (weighted %)		153 (16.8)	8 (22.9)	0.87 (*df*=1)		.35	
Nonwhite race/ethnicity, n (weighted %)		196 (30.6)	9 (32.4)	0.05 (*df*=1)		.83	
College graduate or higher education, n (weighted %)		313 (27.5)	23 (55.9)	13.02 (*df*=1)		<.001	
Married/partnered, n (weighted %)		561 (74.7)	29 (82.4)	1.02 (*df*=1)		.31	
Currently employed, n (weighted %)		389 (61.2)	19 (61.8)	0.01 (*df*=1)		.94	
Annual household income US $60,000 or higher, n (weighted %)		503 (64.6)	22 (61.8)	0.12 (*df*=1)		.73	
**Region of country, n (weighted %)**					3.85 (*df*=3)		.28
	South	338 (47.3)	15 (58.8)				
	West	167 (18.2)	7 (5.9)				
	Midwest	180 (21.8)	7 (20.6)				
	Northeast	95 (12.7)	5 (14.7)				
Nonmetro residence, n (weighted %)		643 (17.9)	28 (8.8)	1.87 (*df*=1)		.17	
**Military branch, n (weighted %)**					2.05 (*df*=4)		.73
	Army	285 (45.5)	13 (41.2)				
	Navy	200 (21.3)	6 (17.6)				
	Air Force	168 (17.6)	8 (23.5)				
	Marine Corps	57 (7.4)	4 (11.8)				
	Other	70 (8.3)	3 (5.9)				
Combat veteran, n (weighted %)		258 (37.4)	13 (41.2)	0.20 (*df*=1)		.66	
10+ years of military service, n (weighted %)		310 (39.6)	13 (35.3)	0.26 (*df*=1)		.61	
**Prepandemic variables**							
	Adverse childhood experiences, weighted mean (SD)	1.8 (2.2)	2.6 (3.0)	2.14		.02	
	Number of traumas, weighted mean (SD)	9.4 (9.2)	12.7 (10.2)	2.05		.02	
	Lifetime MDD^a^ and/or PTSD^b^, n (weighted %)	209 (31.6)	15 (35.3)	0.21 (*df*=1)		.65	
	Lifetime AUD^c^ and/or DUD^d^, n (weighted %)	327 (48.3)	16 (44.1)	0.23 (*df*=1)		.63	
	Extraversion, weighted mean (SD)	3.7 (1.6)	4.9 (1.3)	4.56		<.001	
	Agreeableness, weighted mean (SD)	4.9 (1.4)	5.0 (1.2)	0.26		.40	
	Conscientiousness, weighted mean (SD)	5.8 (1.1)	5.7 (1.4)	0.35		.36	
	Emotional stability, weighted mean (SD)	5.1 (1.4)	5.1 (1.5)	0.07		.47	
	Openness to experiences, weighted mean (SD)	4.8 (1.2)	5.0 (1.1)	0.86		.20	
**Pandemic-related variables**							
	COVID-19 infection to self, n (weighted %)	70 (8.5)	6 (17.6)	3.41 (*df*=1)		.07	
	COVID-19 infection to household member, n (weighted %)	61 (9.0)	4 (15.2)	1.43 (*df*=1)		.23	
	COVID-19 infection to nonhousehold member, n (weighted %)	360 (45.9%)	18 (55.9%)	1.32 (*df*=1)		.25	
	Know someone who died from COVID-19, n (weighted %)	55 (6.2)	3 (5.7)	0.01 (*df*=1)		.91	
	Pandemic-related media exposure, weighted mean (SD)	1.6 (2.0)	2.4 (2.6)	2.30		.01	
	Pandemic-related worries, weighted mean (SD)	–0.1 (1.0)	0.2 (1.1)	1.39		.08	
	Pandemic-related social restriction stress, weighted mean (SD)	0.0 (1.1)	0.3 (1.1)	1.80		.04	
	Pandemic-related financial stress, weighted mean (SD)	0.1 (1.1)	0.2 (1.4)	0.70		.24	
	Pandemic-related worsening of relationships, weighted mean (SD)	0.0 (1.1)	0.5 (1.1)	2.21		.01	
	Pandemic-related PTSD symptoms, n (weighted %)	113 (13.7)	12 (29.4)	6.65 (*df*=1)		.01	

^a^MDD: major depressive disorder.

^b^PTSD: posttraumatic stress disorder.

^c^AUD: alcohol use disorder.

^d^DUD: drug use disorder.

### Characterizing Usage of COVID Coach Explorer

#### Distinct Days of Use

Most participants who downloaded the COVID Coach app used it for 4 distinct days or fewer. Half (17/34, 50%) used the app on exactly 1 day and an additional 24% (8/34) used the app on 2 distinct days, followed by one person who used the app for 3 days, and just over 10% of the sample (4/34, 12%) used the app on 4 distinct days. Distinct days of use ranged from 1 to 16 (mean 2.53, SD 2.86). Retention over the course of the observation period varied slightly more. Among participants who used the app for 2 days or more, the mean number of days retained was 97.41 (SD 97.07) with a range of 1 day (meaning the app was used on 2 consecutive days) to 300 days (ie, the time between the first day of app use and the last day of app use spanned approximately 10 months).

#### Overall Key Content Usage

Nearly half of the participants who downloaded the app (15/34, 44%) completed at least one key event within one of the four sections of the app (Manage Stress, Learn, Mood Check, or Find Resources). The manage stress tools were tried 119 times by 11/34 participants (mean 10.82 tools, SD 19.81; mode 2, range 1-67). The most frequently accessed tool was “Finding Meaning” (12/119, 10.1% of all tool usage). Learn topics were viewed 35 times by 3/34 participants (range 1-67 topics per participant). Within the Mood Check section, 31 assessments were completed by 5/34 unique participants and one participant added a personal goal. Lastly, resources within the Find Resources section were viewed 20 times among 4/34 participants (range 2-14 resources). 

#### Comparison of Veterans Who Used the COVID Coach App Only One Day Versus More Than One Day

Among the 34 veterans who downloaded/used the COVID Coach app, we additionally examined characteristics of veterans who used the app on one day only (n=17) versus 2 or more distinct days (n=17; mean 3.6, SD 2.0) on all of the characteristics shown in Table 2. Results of these analyses revealed that, relative to veterans who used the app one daily only, those who used the app 2 or more days scored lower on prepandemic measures of agreeableness (mean 4.3, SD 1.1 vs mean 5.6, SD 1.0; t_1049_=3.30, *P*=.003; *d*=1.18) and emotional stability (mean 4.54, SD 1.3 vs mean 6.7, SD 1.5; t_1049_=2.24, *P*=.003; *d*=0.80), and higher on measures of pandemic-related financial stressors (mean 0.9, SD 1.8 vs mean –0.2, SD 0.6; t_1049_=2.31, *P*=.03; *d*=0.83) and relationship difficulties (mean 1.0, SD 1.1 vs mean –0.1, SD 0.9; t_1049_=3.02, *P*=.005, *d*=1.08). Usage of the four key content areas within the app (Manage Stress, Mood Check, Learn, Find Resources) on the first day of use did not predict returning to the app for a second day (all *P*>.10).

## Discussion

### Principal Findings

This study is among the first to provide population-level estimates for smartphone ownership among US veterans, identify predictors of uptake and usage for a mental health app focused on pandemic-related stressors, and examine objective app usage data. We found that the vast majority of participants in this study owned smartphones and many had a lifetime history of mental health concerns, yet relatively few individuals were willing to try a free mental health app for pandemic-related stress, and among those who did, overall app usage was fairly low. Among older veterans who completed their KnowledgePanel survey on a mobile device (n=814/3078, mean age 56 years), 91.1% owned smartphones. Smartphone ownership among veterans who completed their survey on a laptop or desktop computer was also high (76.6%) and relatively close to national estimates for the 50-64–year age group [21]. This is notable because the mean age of participants in this group was nearly 65 years, and smartphone ownership estimates for adults aged 65 and older is only 61% of the US population [21]. This finding suggests that members of the KnowledgePanel sample may be more open to adopting mobile technologies than the general population, but it could also suggest that older veterans may be more willing to adopt smartphones than the general older adult population. Additionally, participants who completed their KnowledgePanel survey on a mobile device were more likely to identify as women and nonwhite, be college- or higher-educated, be currently employed, and more likely to have a lifetime history of mental health concerns than those who did not complete their survey on a mobile device. The relationship between mobile device ownership and socioeconomic status is consistent with prior research [21,33]. These findings also highlight that digital health interventions such as mobile apps may be a way to reach older women veterans, veterans of color, and older veterans with mental health concerns.

The results further revealed that smartphone adoption may not necessarily equate to mobile mental health uptake and usage. Among the 800 participants who were offered the opportunity to download and try COVID Coach, only 34 (3.3%) downloaded and tried the app, 50% of whom (n=17/34) returned to the app after their first day of use, and 44% (n=15/34) engaged with content in at least one of the four key content areas within the app. Nonetheless, there were some encouraging findings in the data. Although only 3.3% of eligible participants downloaded and tried the app, one of the strengths of utilizing the KnowledgePanel sample is it being comprised of a population-based, nationally representative sample of US adults. Using population benchmarks, if 3.3% of the US veteran population were to download an app for mental health, that would equate to more than 600,000 veterans. Thus, the potential reach for an app is large, and even if only some veterans use the app for an extended period of time, that may potentially translate to thousands of users and ultimately an important public mental health impact [56]. It is important to note that the majority of veterans do not use the VA as their primary source of health care, and veterans that do use the VA are more likely to be black, younger, female, unmarried, have lower household incomes, and have a lifetime history of psychopathology [57]. Therefore, a multipronged approach is needed to help promote app awareness and uptake among veterans being served both outside and within the VA. For example, national communication strategies that reach veterans wherever they are, such as features in popular media [58,59], promotion from organizations that serve veterans (eg, [60]), virtual veteran communities (eg, Women Veterans Network [WoVEN] [61]), resources and tools for community-based providers (eg, Community Provider Toolkit [62]), and social media campaigns that are tailored to specific veteran communities may be best suited to reach veterans not receiving care within the VA. Programs such as Tech Into Care [63], which train a wide variety of VA staff, including doctors, nurses, psychologists, social workers, audiologists, and chaplains, to be mobile health (mHealth) ambassadors and spread the word about apps for mental health may help raise awareness among both VA employees and the veterans they serve. mHealth ambassadors, who are trained in how to use, offer, and implement mobile mental health apps, may also help develop dissemination approaches that take veteran characteristics, local or regional factors, and Veterans Integrated Services Networks context into account.

Additionally, veterans who downloaded COVID Coach reported a greater number of adverse childhood experiences, greater extraversion, and greater severity of pandemic-related PTSD symptoms (ie, exaggerated startle response) than those who did not download the app. Furthermore, among those who did download the app, veterans with lower levels of emotional stability and who experienced greater pandemic-related financial stressors and relationship difficulties were more likely to return to the app for a second day of use than those who only used the app for a single day. Because COVID Coach was specifically designed to provide tools and resources for coping with pandemic-related stress and concerns, the app was preferentially downloaded by veterans in the sample with greater mental health needs during the pandemic. Indeed, the interactive coping tools in the Manage Stress section were the most popular among veterans who utilized the app, a finding that is consistent with previous work [31]. The “Finding Meaning” tool was the most frequently utilized coping tool within the app. This is notable and contrasts with usage among the general population where “Ambient Sounds,” the first tool in the list due to alphabetization, was the most utilized tool. Among older adults, finding purpose and meaning in life is associated with better health outcomes, including cognitive health [64,65]. Digital mental health interventions that target helping older adults cultivate purpose and meaning in life could have an important impact on mental health as well as promote cognitive health.

A notable strength of this study is that it analyzed data from a contemporary, nationally representative, probability-based sample of US veterans. We were able to estimate the prevalence of smartphone ownership, as well as closely examine the characteristics of veterans who did and did not download the COVID Coach mobile mental health app during the height of the COVID-19 pandemic. Furthermore, we were also able to explore veteran characteristics associated with app download and app usage, which were measured automatically via captured analytics data. Collectively, the results of this study provide an important contribution to understanding veteran smartphone ownership rates, characteristics associated with downloading (or not downloading) an app for mental health, and predictors of return app usage.

### Limitations

There are several key limitations of this study. First, the opportunity to download the COVID Coach app was presented as the last screen of a survey with a median completion time of nearly 32 minutes. Following survey completion that assessed a broad range of factors, including pandemic stressors and psychiatric symptoms, participants may have been fatigued and reluctant to take on yet another optional task, particularly since downloading and trying the app did not impact their study compensation. Second, the app was only offered to veterans who completed the survey on a mobile device, regardless of whether they owned a smartphone or tablet, thus limiting the potential number of potential app downloads. Nearly two-thirds of the sample did not complete their survey on a mobile device, yet the vast majority of those participants (76.6%) indicated that they did own a smartphone. Thus, participants who may have been interested in exploring the app were never offered the chance to download it. Third, for those who did receive the information about how to download COVID Coach, the perceived benefits and utility could have been specified more clearly. Previous research has indicated that older veterans, especially those living in rural areas, may be less likely to see the benefits of using mobile apps [26]. It is possible that download instructions were not sufficiently clear and the rationale for downloading the app was not sufficiently compelling for some individuals. Further qualitative work would be helpful in identifying how to optimally present this information to encourage high uptake of the app. Finally, we only have objective use data to understand engagement with COVID Coach. More in-depth qualitative information is needed to explore older veterans’ experiences with the app, including usability of the design, appropriateness of the content, and other factors that may have influenced if, when, and how often they used the app.

### Future Research Directions

Results of this study underscore the importance of research that addresses the needs and preferences of veterans to help ensure that the tremendous digital health innovation fueled by the pandemic does not reinforce or exacerbate existing inequities [66]. Digital health tools, including apps, can be part of the solution to help promote better mental health and health care outcomes for older adults [67], as long as they address fundamental issues of digital health equity, such as digital health literacy and inclusive design [68]. To best meet the needs of older veterans, and older adults more generally, future research should utilize qualitative methods and co-design processes to ensure that interventions are solving mental health challenges in usable, meaningful, and engaging ways. Co-design can help address barriers that are specific to older adults (eg, [69]); ensure that the product is findable, accessible, usable, desirable, credible, useful, and valuable (eg, [70]); improve the overall quality of the product [71]; and promote equity and inclusion (eg, [72,73]). More research focused on effective dissemination and implementation strategies is also needed. The number of veterans receiving their health care from the VA has increased over time, and those receiving their care within VA tend to be from populations that are more likely to experience health disparities [57]. These historically underserved groups may benefit from additional digital health supports, and receiving their care within an integrated health care system could potentially facilitate the dissemination and implementation of digital health resources. However, one of the biggest barriers to app uptake identified among veterans receiving care within the VA is app awareness [40]. Furthermore, many veterans receive part or all of their health care outside the VA system. Developing effective strategies for raising awareness of mental health apps and facilitating their usage are crucial for adoption and in turn increased impact.

### Conclusions

The COVID-19 pandemic has accelerated the creation and use of digital mental health resources across a variety of settings. As the veteran population in the United States is aging and their smartphone ownership is growing, they are also more successfully engaging with digital health products [23]. To our knowledge, this study is one of the first to document current smartphone ownership rates in the US veteran population, the majority of whom were older, and to examine predictors of uptake and usage of a mental health app focused on COVID-19–related stressors. Although adoption of COVID Coach was relatively low, the app was more likely to be utilized by individuals facing pandemic-related stressors and associated psychiatric symptoms, suggesting that apps may be a way to reach veterans with mental health needs during the pandemic and beyond.

Collectively, results of this research suggest that mental health apps have the potential to reach a significant minority of older veterans, although continued efforts are needed to identify strategies to bolster uptake in more naturalistic settings. More work is needed to ensure uptake and meaningful engagement with mental health apps. As the pandemic continues to impact mental health globally, digital mental health resources have an important role to play in meeting the needs of veterans, and the general population, during the pandemic and beyond.

## Appendix

Multimedia Appendix 1 COVID Coach Explorer screenshots.

## Abbreviations

GAD-7: Generalized Anxiety Disorder-7
JSON: JavaScript object notation
mHealth: mobile health
NHRVS: National Health and Resilience in Veterans Study
OR: odds ratio
PCL-5: PTSD Checklist for DSM-5
PHQ-9: Patient Health Questionnaire-9
PTG: post traumatic growth
PTSD: posttraumatic stress disorder
VA: US Department of Veterans Affairs
WoVEN: Women Veterans Network
