# Supplementary material for: Predicting Uptake of the COVID Coach App Among US Military Veterans: Funnel Analysis Using a Probability-Based Panel
Source: JMIR Ment Health. 2022 Apr 5;9(4):e36217. doi: 10.2196/36217 (PMC8985691; doi:10.2196/36217)

COVID Coach Home Screen

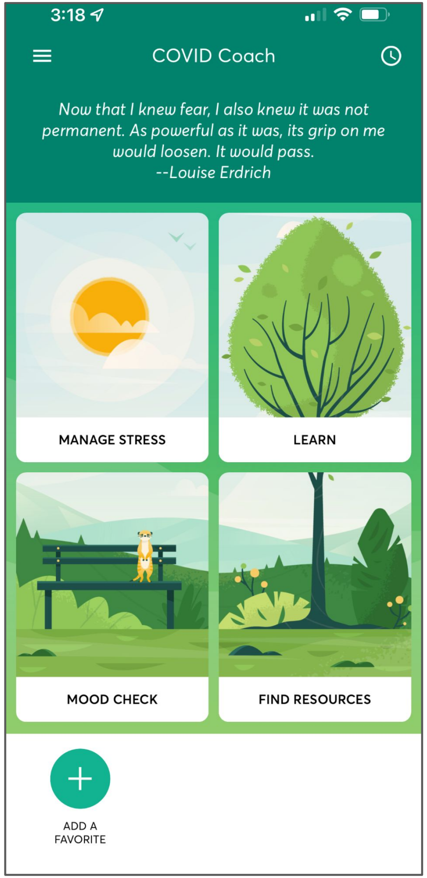

Manage Stress Overview

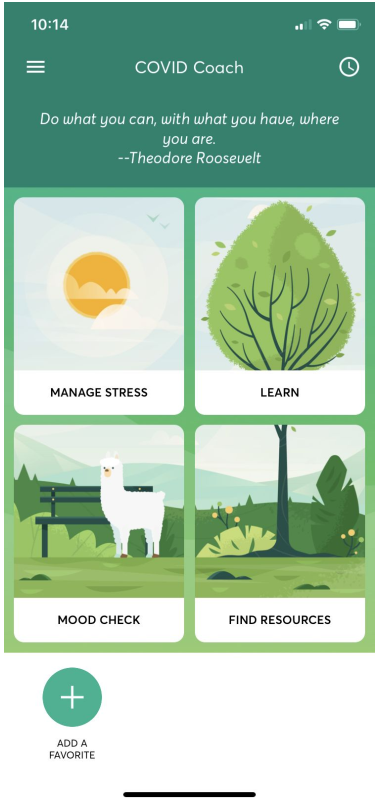

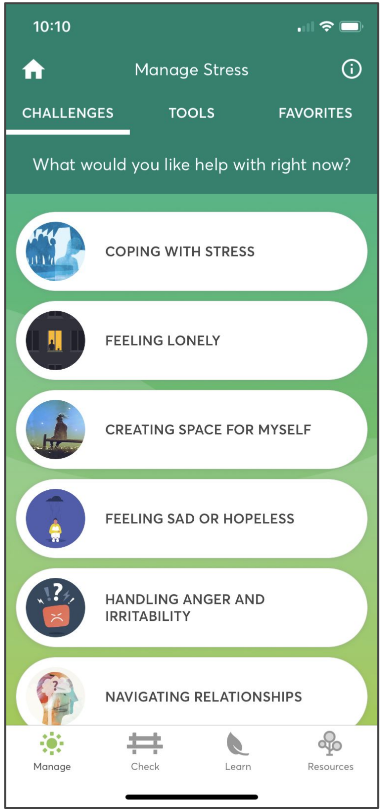

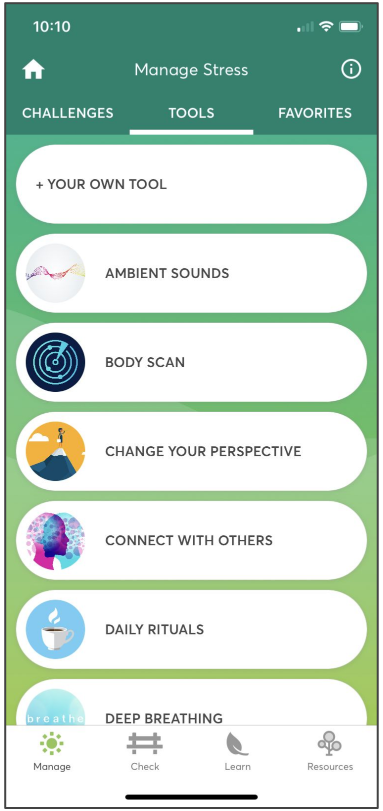

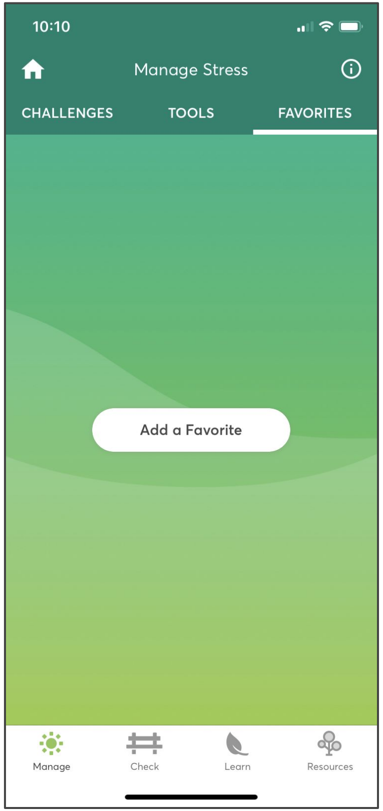

Manage Stress: Coping Tool Example 1

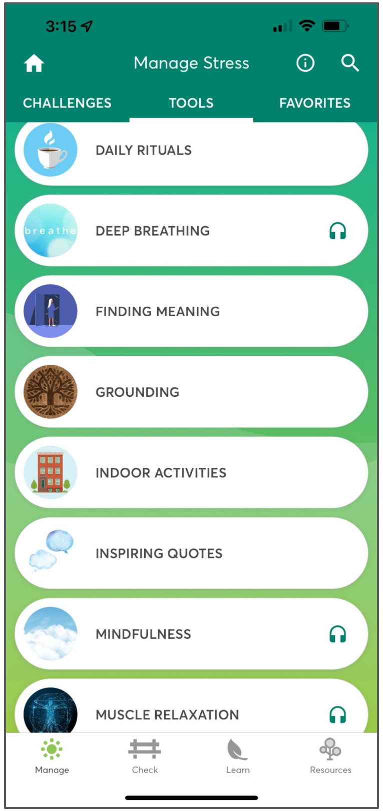

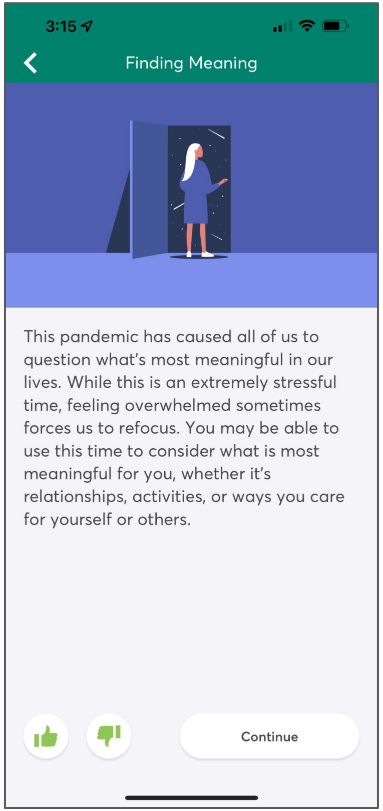

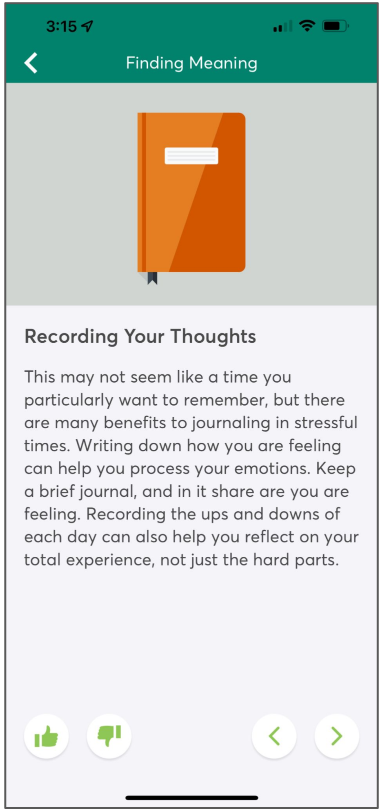

Manage Stress: Coping Tool Example 2

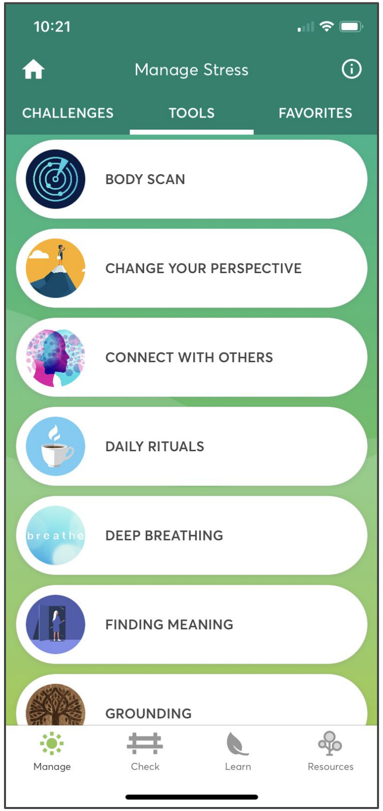

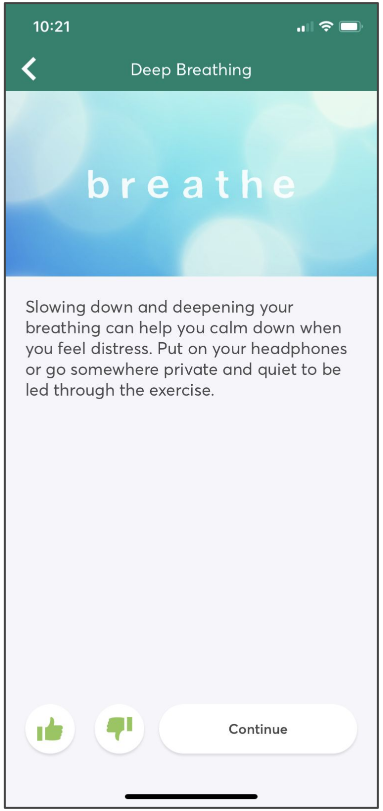

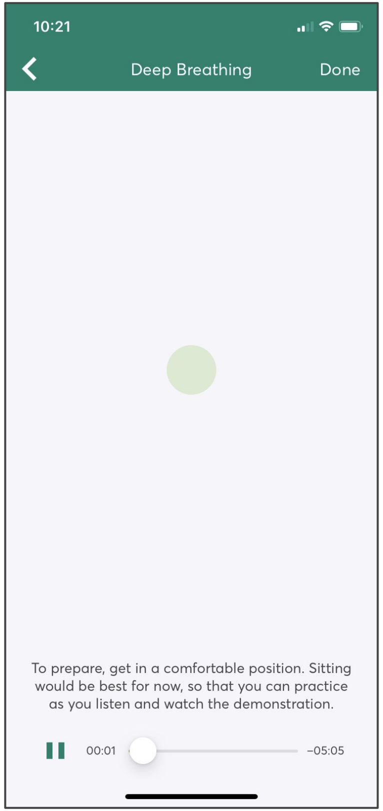

Learn Overview

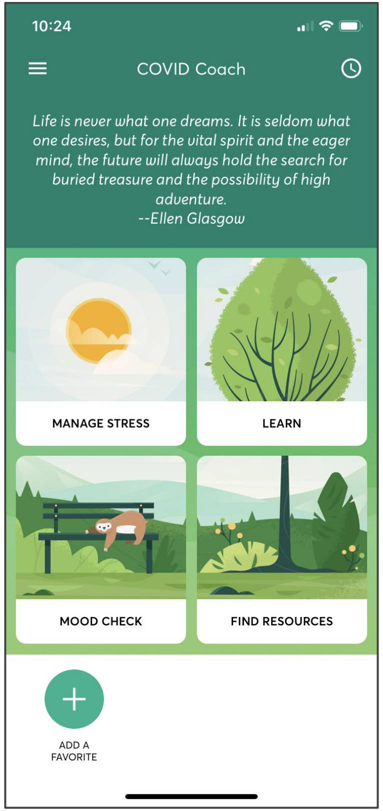

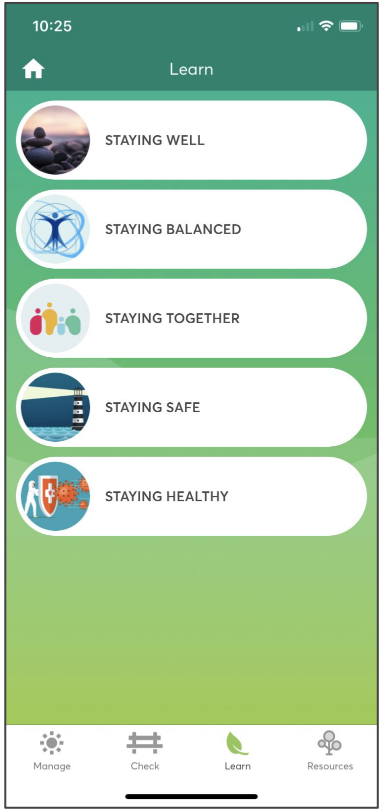

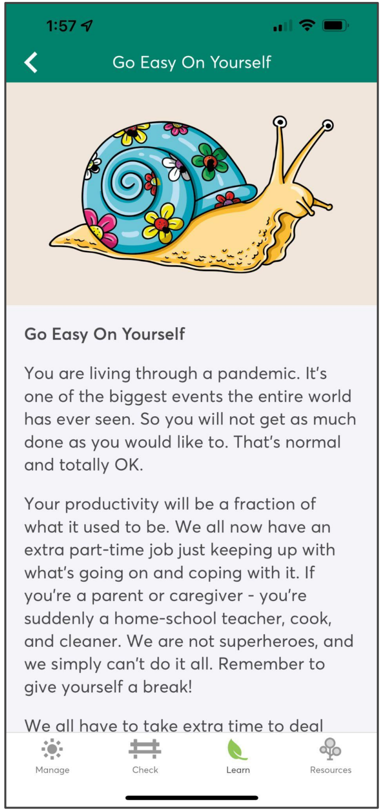

Learn > Sub-sections

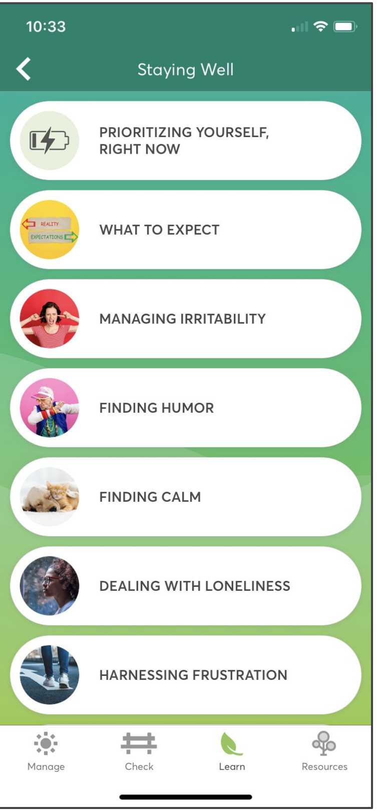

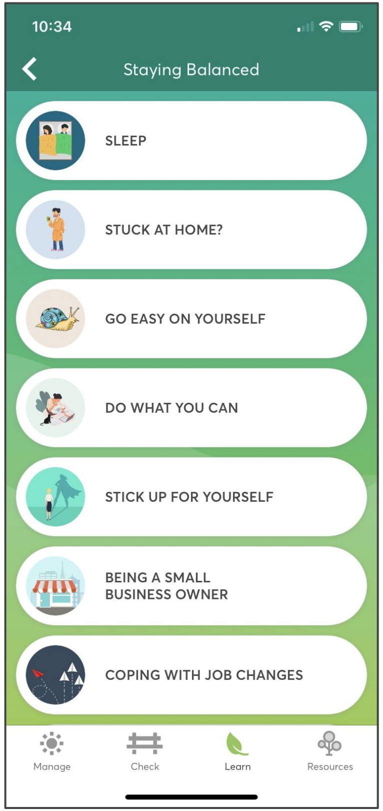

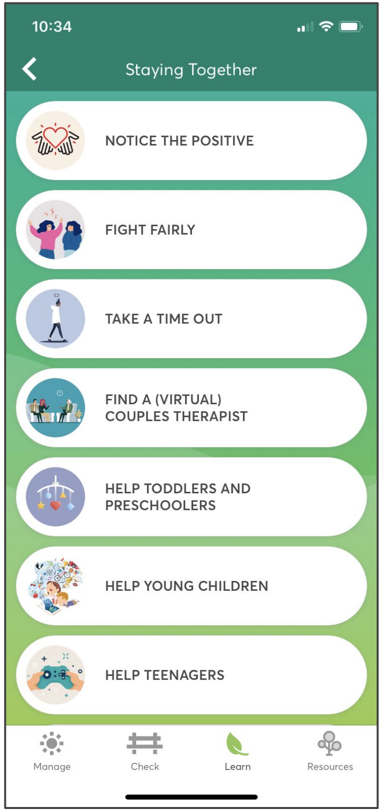

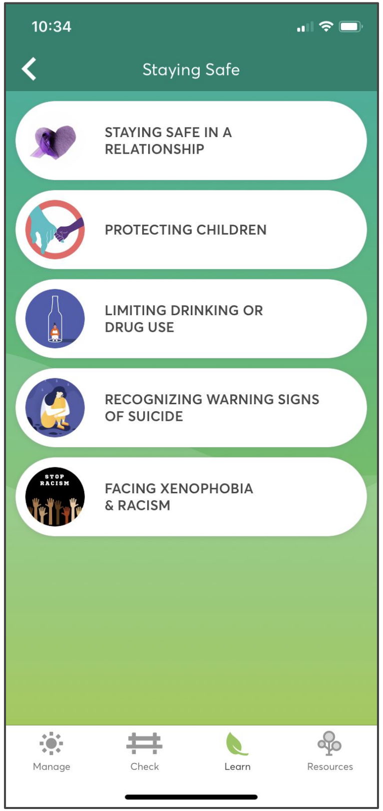

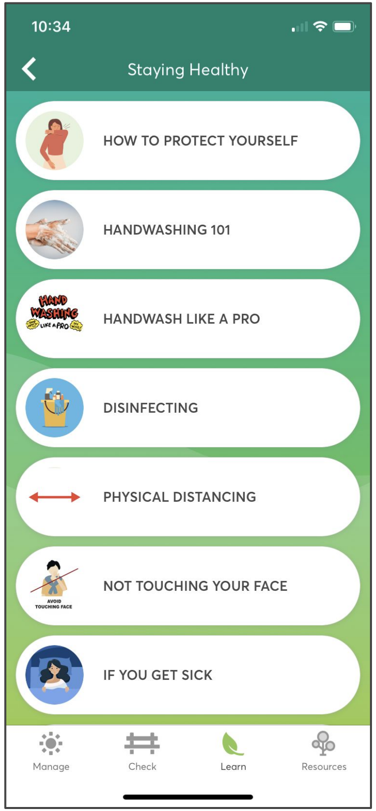

Mood Check Overview

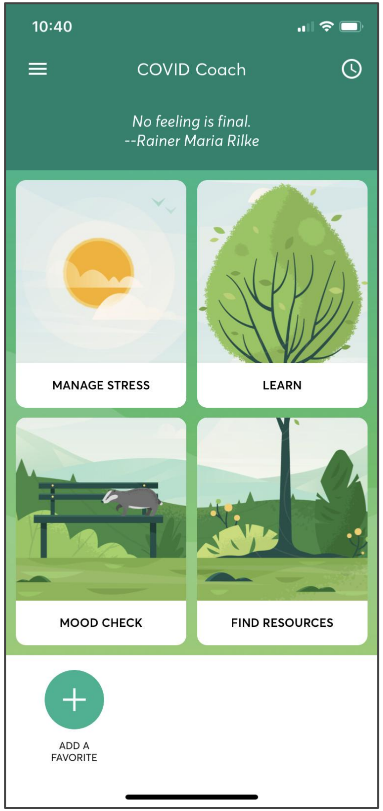

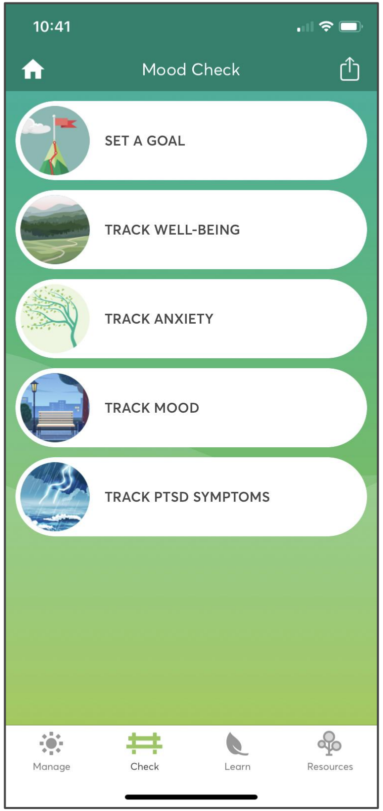

Mood Check: Set A Goal

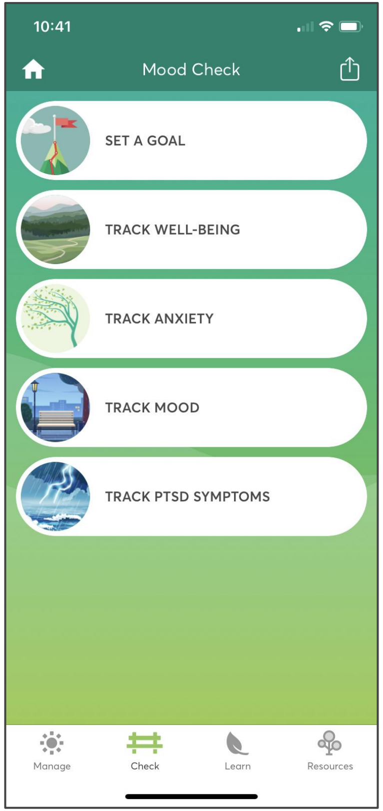

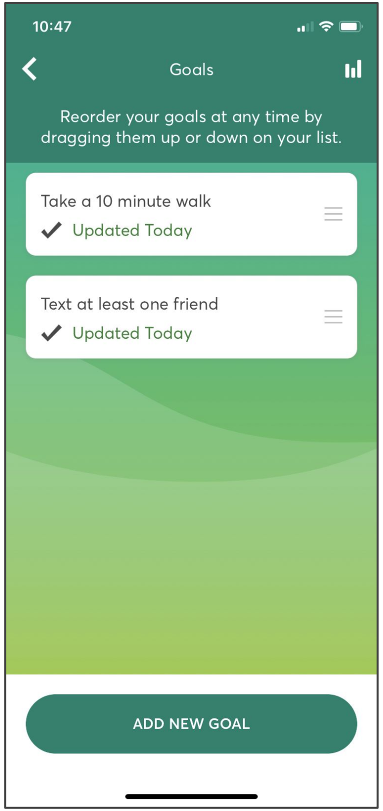

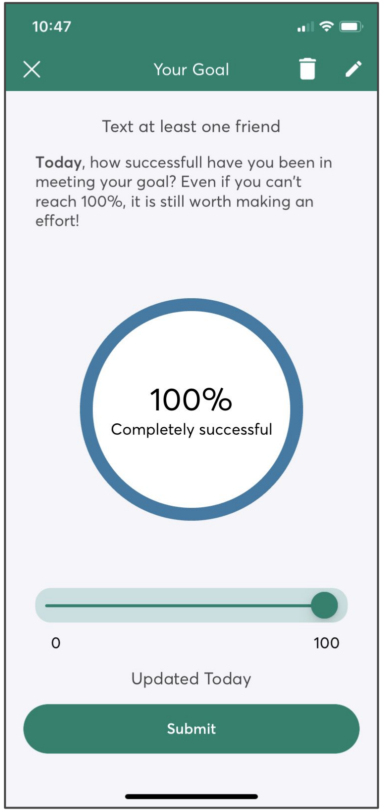

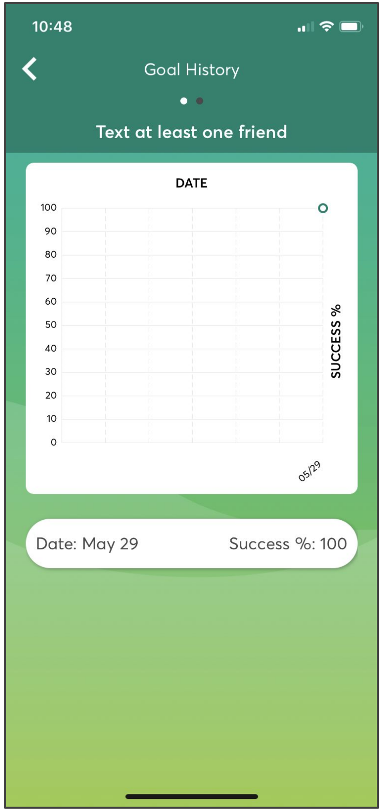

Mood Check: Track Well-Being (WEMBWS)

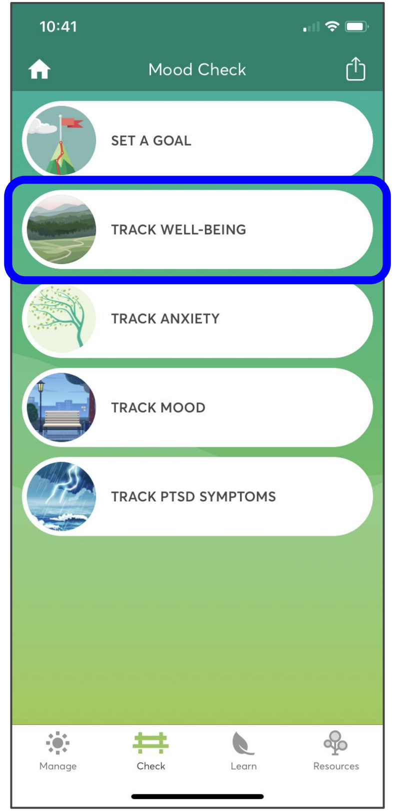

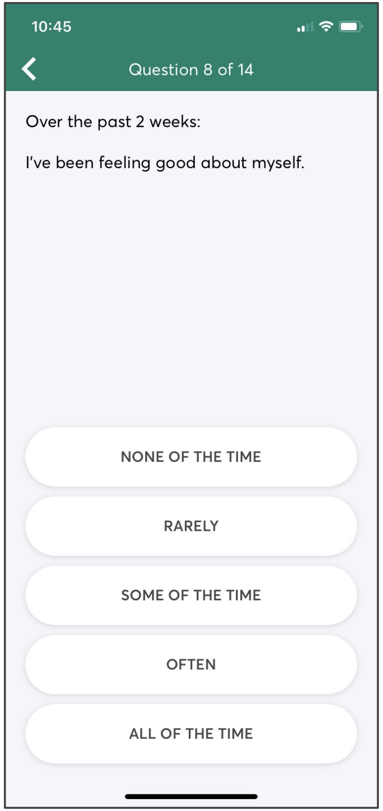

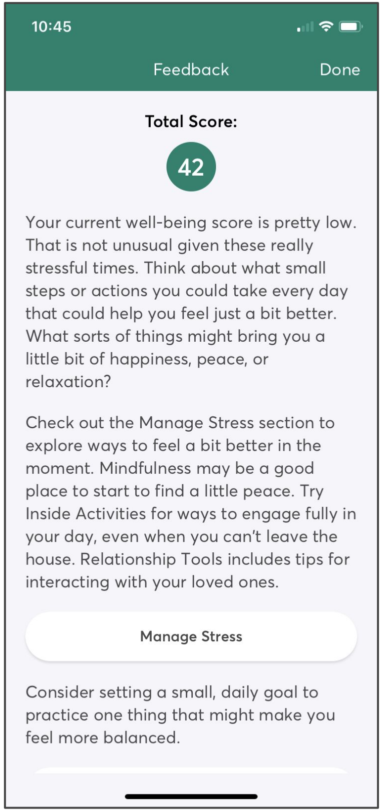

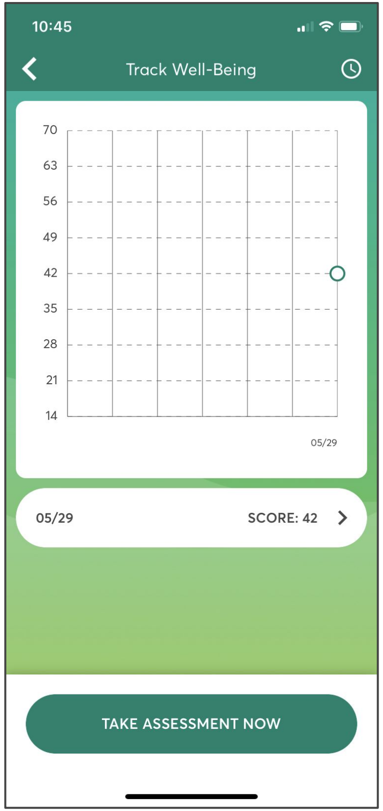

Find Resources Overview

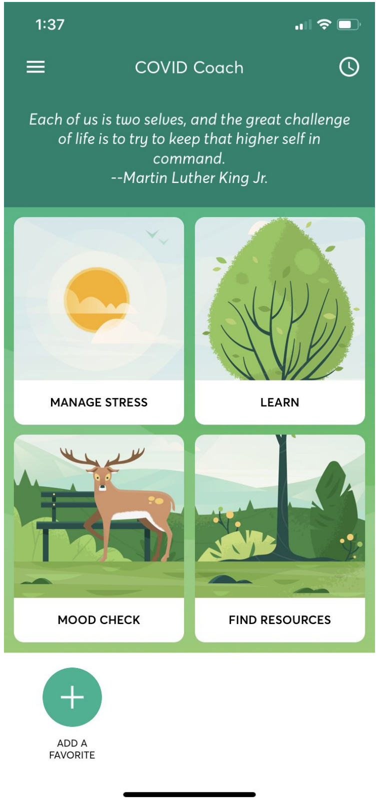

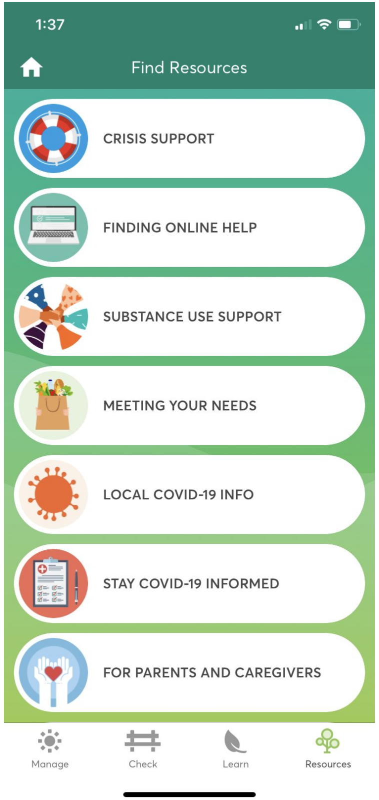

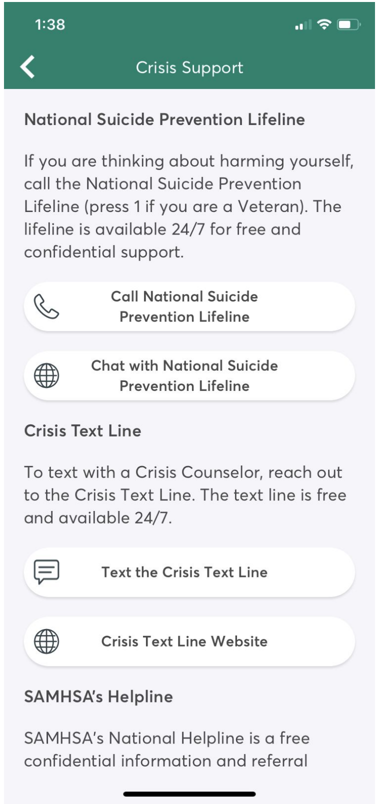

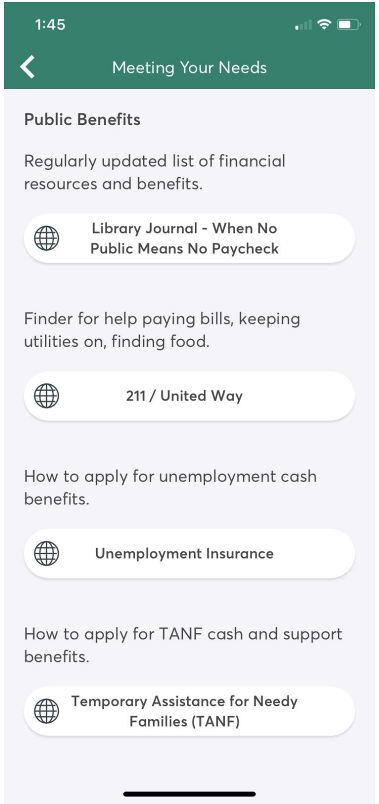

Supplement: Multimedia Appendix 1 [file mental_v9i4e36217_app1.pdf]
